# An advanced glioma cell invasion assay based on organotypic brain slice cultures

**DOI:** 10.1186/s12885-018-4007-4

**Published:** 2018-01-30

**Authors:** Tanja Eisemann, Barbara Costa, Jens Strelau, Michel Mittelbronn, Peter Angel, Heike Peterziel

**Affiliations:** 1 Division of Signal Transduction and Growth Control, DKFZ/ZMBH Alliance, Heidelberg, Germany; 2 Functional Neuroanatomy, University of Heidelberg, Heidelberg, Germany; 3 Institute of Neurology (Edinger-Institute), University Hospital Frankfurt, Goethe University, Frankfurt, Germany; 4 Luxembourg Centre of Neuropathology (LCNP), Dudelange, Luxembourg; 5 Laboratoire National de Santé, Dudelange, Luxembourg; 6 Luxembourg Centre for Systems Biomedicine (LCSB), University of Luxembourg, Esch-sur-Alzette, Luxembourg; 7 Department of Oncology, NORLUX Neuro-Oncology Laboratory, Luxembourg Institute of Health (L.I.H.), Strassen, Luxembourg; 8 Present address: Translational Program, Hopp Children’s Cancer Center at NCT Heidelberg (KiTZ), University Hospital and DKFZ Heidelberg, Heidelberg, Germany; 9 Present address: Clinical Cooperation Unit Pediatric Oncology, DKFZ, Heidelberg, Germany; 10 German Consortium for Translational Cancer Research (DKTK), Heidelberg, Germany

**Keywords:** migration, organotypic brain slices, tumor microenvironment, glioblastoma, three-dimensional invasion assay

## Abstract

**Background:**

The poor prognosis for glioblastoma patients is caused by the diffuse infiltrative growth pattern of the tumor. Therefore, the molecular and cellular processes underlying cell migration continue to be a major focus of glioblastoma research. Emerging evidence supports the concept that the tumor microenvironment has a profound influence on the functional properties of tumor cells. Accordingly, substantial effort must be devoted to move from traditional two-dimensional migration assays to three-dimensional systems that more faithfully recapitulate the complex in vivo tumor microenvironment.

**Methods:**

In order to mimic the tumor microenvironment of adult gliomas, we used adult organotypic brain slices as an invasion matrix for implanted, fluorescently labeled tumor spheroids. Cell invasion was imaged by confocal or epi-fluorescence microscopy and quantified by determining the average cumulative sprout length per spheroid. The tumor microenvironment was manipulated by treatment of the slice with small molecule inhibitors or using different genetically engineered mouse models as donors.

**Results:**

Both epi-fluorescence and confocal microscopy were applied to precisely quantify cell invasion in this ex vivo approach. Usage of a red-emitting membrane dye in addition to tissue clearing drastically improved epi-fluorescence imaging. Preparation of brain slices from of a genetically engineered mouse with a loss of a specific cell surface protein resulted in significantly impaired tumor cell invasion. Furthermore, jasplakinolide treatment of either tumor cells or brain slice significantly reduced tumor cell invasion.

**Conclusion:**

We present an optimized invasion assay that closely reflects in vivo invasion by the implantation of glioma cells into organotypic adult brain slice cultures with a preserved cytoarchitecture. The diversity of applications including manipulation of the tumor cells as well as the microenvironment, permits the investigation of rate limiting factors of cell migration in a reliable context. This model will be a valuable tool for the discovery of the molecular mechanisms underlying glioma cell invasion and, ultimately, the development of novel therapeutic strategies.

## Background

Glioblastoma is the most frequent and malignant primary brain tumor, with a median survival of 12–15 months after diagnosis. Despite extensive surgical resection, chemo-, and radiotherapy, glioblastoma is still considered incurable [1–3]. The diffuse infiltration of tumor cells into adjacent healthy brain tissue is a major cause of treatment failure, and so the characterization of signaling pathways and effector molecules that drive glioblastoma invasion is a major aim in glioblastoma research (for reviews see [4, 5]).

Most studies of tumor cell migration involve simple and inexpensive two-dimensional methods like the in vitro scratch and Boyden chamber/transwell assays. However, recent studies have shown striking differences in protein functions in two- and three-dimensional contexts [6–8]. Furthermore, in vivo tumor cells are embedded in a three-dimensional matrix consisting of the extracellular matrix (ECM) and multiple cell types, which can all interact with tumor cells. Emerging evidence highlights the substantial impact of these reciprocal interactions within the tumor microenvironment on tumor cell invasion [9], and therefore the requirement for an invasion assay that closely mimics the environmental milieu that glioma cells encounter in vivo. Invading glioblastoma cells follow distinct anatomical features called Scherer’s structures. These include meninges and the subjacent subarachnoid space, blood vessels, myelinated nerve fibers and the extracellular space between neuronal or glial processes in the brain parenchyma [10]. Taking into account that glioblastoma cells migrate along these pre-existing multicellular structures - that cannot simply be mimicked by co-cultivation of the relevant cell types - we used organotypic murine brain slice cultures as a three-dimensional invasion matrix. Preserving essential features of the host tissue such as neuronal connectivity, glial-neuronal interactions and an authentic ECM, organotypic brain slice cultures have mainly been used to study developmental, structural and electrophysiological aspects of neuronal circuits (for reviews see [11, 12]). Previously, these organotypic cultures have also been presented as a novel tool to examine the migratory behavior of ex vivo implanted tumor cells [13–16]. However, the reported methods were based on human brain slices, or the extent of invasion observed was rather low and did not reflect the high infiltration capacity of glioblastoma cells in vivo. Here, we present an optimized and reproducible protocol to assess highly infiltrating glioma cells in an adult murine brain slice. In particular, we show that the usage of a membrane dye with red-shifted fluorescence spectra and tissue clearing results in greatly increased image quality. Finally, we present a selection of application examples, including the treatment of tumor cells or the manipulation of the tumor cell environment by pharmacological inhibitors and the use of genetically modified mice as brain slice donors. Knowledge gained from in vitro and high-throughput approaches can be functionally validated by this method, accentuating its value as link between in vitro and animal studies.

## Methods

### Preparation of brain slices

6–8 week old C57Bl/6 wild-type or knockout mice were euthanized, the brain was isolated and the cerebellum removed with a scalpel. Using insect forceps the brain was transferred to the vibratome (Leica VT1200 S) platform and immediately fixed to this device by applying a drop of superglue. The lateral short side of the brain was placed facing the blade, in order to reduce mechanical stress. 350 μm thick coronal slices were cut with a maximal speed of 0.2 mm/s. Up to three slices were gathered per filter (Millipore #PICM03050). The transfer of the slices was facilitated by a brush and addition of brain slice medium on top of the filter. The brain slice medium is composed of MEM (Sigma # M2279), 25% heat-inactivated horse serum (Life Technologies # 26050070), 25 mM HEPES (Sigma # H0887-100 mL), 1 mM L- glutamine (Sigma, # G7513), 5 mg/ml glucose (Sigma # G8769), 100 U/ml penicillin/streptomycin (Sigma # P4333). For cultivation at 37 °C and 5% CO_2_ the medium was removed from the filter and 1 ml of fresh brain slice medium was added below. The medium was refreshed after 18-24 h and then every other day. Brain slices were cultivated air-exposed. To prevent dehydration the tissue was moistened with a drop of medium every day, and remaining excess medium removed. Although the brain slices can be cultivated for at least one week, experiments were performed at d2 and, due to the high migratory capacity of glioma cells, terminated on d4.

### Preparation of fluorescently labeled spheroids

Murine (SMA560) and human (LN319; U87MG; U251MG) glioma cell lines cultivated in serum-containing medium (DMEM, Sigma # D5671; 10% FBS, Sigma # F7524; 2 mM L-glutamine) were trypsinized and counted. 1 × 10^6^ cells/ml PBS were incubated with 5 μl lipophilic dye DiD (1 mg/ml in DMSO, Biotium #60014) or 5 μl DiI (Biotium # 30022) for 30 min at 37 °C. After two washing steps 500 cells/well were seeded in a flat-bottom 96-well plate coated with 50 μl low melt agarose (Genaxxon # M3049.0010; 1% in PBS). SMA560 and U87MG spheroids were cultivated in serum-containing DMEM medium, whereas the spheroid formation of LN319 and U251MG required cultivation in serum-free neurobasal medium (Thermo Fisher Scientific # 10888022) containing B27 supplement (Thermo Fisher Scientific # 17504044), 20 ng/ml of both EGF (Promokine # C-60170) and FGFb (Promokine # C-60240), 2 μg/ml heparin sodium salt (Sigma # H3149), 2 mM L-glutamine and 1% penicillin/streptomycin (p/s; 100 U/ml).

Human and murine primary cells were cultivated as spheroids in serum-free medium. Primary human cells were kept in neurobasal medium, and primary murine cells were cultivated in DMEM/F12 (Thermo Fisher Scientific # 21331020) medium containing N 2 supplement (Life Technologies # 17502048), 20 ng/ml of both EGF and FGFb, 2 mM L-glutamine and 1% penicillin/streptomycin (100 U/ml). To generate spheroids for subsequent implantations, cells were labeled as described above and seeded in agarose-free U-bottom 96-well plates (500 cells/well; Greiner # 650185).

Most glioma cell lines and primary cells we tested formed spheroids under the described conditions. However, in some cases the protocol might have to be adjusted to cultivation in neurobasal medium, or to the hanging drop culture protocol. The last cultivation technique requires medium supplementation with 20% methyl cellulose. The single cell suspension is pipetted in drops of 20 μl on the inside of a dish which is then slowly inverted.

For generation of heterotypic spheroids, astrocytes were isolated from 3 day old neonatal mice. Briefly, brains were isolated and the meninges removed. Cortical tissue was ground on a 70 μm cell strainer with a glass pestle. After three washes, cells were cultured in DMEM (10% FBS, 2 mM L-glutamine and 1% p/s) on poly-lysine-coated culture vessels. After a cultivation period of at least 10 days astrocytes were used for co-implantation experiments. Astrocytes were labeled with DiI and co-cultivated as heterotypic multicellular spheroids with DiD labeled murine SMA560 glioma cells line in a ratio of 3:2 in agarose coated 96-well plates for two days.

Due to the different growth rates of the tumor cells used in the experiments and the difficulty of precisely measuring the number of cells in an established spheroid, we seeded a fixed number of 500 cells and implanted the tumor cell spheroids when they reached a diameter of approximately 150 μm.

The murine cell line SMA560 was provided by Prof. Michael Platten (Department of Neurology, University Heidelberg). The human cell lines LN319 and U251 were provided by Prof. Wolfgang Wick (University Hospital Heidelberg), and Prof. Michael Weller (Department of Neurology, University Zurich) provided the human cell line U87MG. Human and murine primary glioblastoma cultures were generated in our laboratory.

### Tumor cell and brain slice treatment with jasplakinolide

500 DiD labeled SMA560 glioma cells were seeded in spheroid-forming conditions per well of a 96-well plate. 18 h prior to implantation, spheroids or brain slices were treated with 1 μM jasplakinolide (Cayman #11705) or DMSO (Sigma # 41639-100ML). 24 h after implantation, brain slices were fixed and imaged by confocal microscopy.

Cell viability in vitro was measured with trypan blue staining (Sigma # 93595-50ML).

### Spheroid implantation

**Fig. 1:**
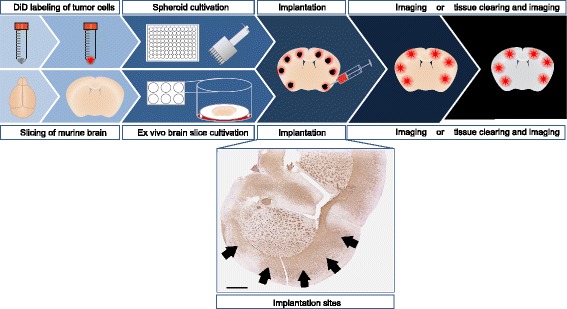
Work flow of the ex vivo invasion assay based on organotypic brain slice cultures. Schematic representation of the individual protocol steps. Immunohistochemistry staining shows MBP pattern of an adult murine brain slice cultivated for four days, arrows indicate implantation sites, scale bar 1 mm

8–10 spheroids per brain slice were manually implanted using a blunt Hamilton syringe (701 N; 10 μl; 26 s/51/3) and a binocular microscope. For this purpose, a single spheroid was aspirated in a maximum volume of 0.5 μl and implanted by release of the total volume into the tissue. This procedure was repeated until 8–10 spheroids were implanted along the cortex (see Fig. 1 for implantation sites). There are several factors that are essential for maximum invasion. Most important in this context is proper implantation of the spheroids. It might help to completely penetrate through the tissue to the filter to estimate its depth; however, it is crucial to not release the spheroid onto the filter. The spheroid must be implanted within the tissue, as release of the spheroid below or on top of the brain tissue will not result in tumor cell invasion but in proliferation or in some cases in collective migration along the tissue surface. Furthermore, tissue integrity is an essential factor for correct implantation. Dehydration of brain slices impedes penetration of the tissue with the needle tip, as the tissue surface becomes too rigid. Conversely, excessive immersion of the brain slice in medium results in tissue degeneration and disintegration upon penetration with the needle tip. Thus, we recommend moistening the brain slices every other day, followed by removal of excessive medium. If these instructions are carefully followed, a successful implantation rate of at least 80% can be achieved.

Following implantation, medium was refreshed and the slices cultivated at 37 °C and 5% CO2. Experiments were terminated 2d after implantation unless otherwise stated. For fixation brain slice medium was removed and 1 - 2 ml 4% PFA added on top of the filter for 2 h at RT or o/n at 4 °C. Fixed slices were transferred with a spatula from the filter into a new 6-well plate containing 2 ml PBS/well. Although the slices can be stored in the parafilm-sealed plate for at least three months in the dark at 4 °C, slices were imaged by epi-fluorescence or confocal microscopy as soon as possible.

### Tissue clearing

To obtain high quality epi-fluorescence microscopy images, autofluorescent brain slices were cleared according to the SeeDB protocol [17]. After incubation in 20% clearing solution for 4-8 h in the dark with gentle shaking, the clearing solution was changed to 40% and subsequently 60% clearing solution each for 4-8 h. Slices were sequentially incubated in 80%, 100% and 115% clearing solution o/n. As clearing solutions of 100% and 115% tend to crystalize, incubation steps were performed in a humid chamber. Cleared slices were stored in the 115% clearing solution in a dark humid chamber at RT. All clearing solutions contain 0,5% α-thioglycerol (Sigma # M1753) and the above given wt/vol percentage of (D-) fructose (Sigma # F0127).

### Imaging and quantification of invasion

For epi-fluorescence imaging the slices were kept in 6-well plates containing PBS or, if the slices had been cleared, 115% clearing solution. For confocal imaging slices were transferred with a spatula onto an object slide and loosely covered with a coverslip.

Z-stack images were transformed to a maximum projection image by using ImageJ [18]. Image quality was optimized by adjusting brightness, contrast and gamma. Migratory cells were visible as spikes emerging from the bulk of the spheroids that had been formed by cells establishing an infiltration path. These invasion sprouts were traced from the center of the mass to the tip using the freehand tool. The radius of the spheroid body (if not determinable spheroid body radius from d0) was subtracted from the measured sprout length. Subsequently, we calculated the average cumulative sprout length by adding up the length of all sprouts of a spheroid and dividing this sum by the number of analyzed spheroids. This statistic integrates sprout length and the number of sprouts to estimate the migratory capacity of the cells. Calculation of the cumulative sprout length is a common tool in angiogenesis research, where it is used as reliable quantification of cell movement and proliferation in a three-dimensional environment [19–22].

### Statistical analysis

Welch’s t-test was performed to evaluate the difference between the cumulative sprout lengths of experimental groups. Differences in the grade of invasion were considered significant if *p* < 0.05. Bonferroni correction of *p*-values was applied for multiple comparisons (in particular, comparison of jasplakinolide treated cells, cells implanted in jasplakinolide treated slices and control cells).

### Immunohistochemistry

4d after brain slice preparation the tissue was fixed and pre-embedded in 2% agar (Carl Roth # 5210.3)- 2.5% gelatin (Merck Millipore # 1040700500; in PBS) without sponge pads and subsequently processed for paraffin embedding. 4-6 μm thick sections were stained according to standard immunohistochemistry protocols. The antibodies used were specific for: glial fibrillary acidic protein (GFAP) (Biolegend # 644701; diluted 1:500), laminin (Progen Biotech # 10765; diluted 1:200), myelin basic protein (MBP) (Abcam # 7349; diluted 1:200) and ionized calcium-binding adapter molecule 1 (Iba1) (Wako # 019–19,741; diluted 1:500).

## Results

### Adult slice cultures retain cytoarchitecture of the brain

**Fig. 2:**
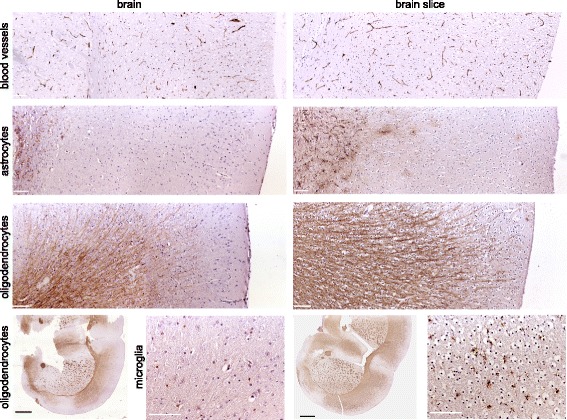
Organotypic brain slice cultures maintain characteristic features of adult brain tissue. Immunohistochemical staining of 4-6 μm sections of adult murine brain slices (prepared from 350 μm vibratome sections and cultivated for four days) compared to 4-6 μm sections of whole adult murine brains. Note the comparable patterns of blood vessels, as indicated by laminin, and myelinating oligodendrocytes, as indicated by MBP staining. We observed slight activation of astrocytes (GFAP) and microglia (Iba1) within the brain slice, presumably induced by the mechanical trauma of vibratome cutting. White scale bars 100 μm, black scale bars 1 mm

The majority of previous publications utilized brain slices from perinatal donors that show a high degree of resistance to mechanical trauma during slice preparation [23]. However, as high grade gliomas are most common among adult patients, we used adult mice with a fully developed brain, including completed myelination, as donors for the preparation of brain slices. To determine whether the cytoarchitecture is preserved, we performed immunohistochemical staining on adult brain slices embedded 4 days after slicing. We observed that blood vessels and myelinated fiber tracts were present and morphologically intact; astrocytes (GFAP) and microglia (Iba1) were slightly activated within the brain slice, presumably induced by the mechanical trauma of cutting (Fig. 2). In contrast to the survival of astrocytes, microglia and endothelial cells, neuronal survival in brain slices has been reported as a major challenge, especially for slices prepared from adult donors [24]. This is partly attributed to the fact that neuronal cell death is induced by axotomy during the process of tissue slicing. However, the structure of myelinated nerve tracts remains intact and provides the same structural surfaces glioma cells encounter in vivo. Taken together, we confirm that the cytoarchitecture of the adult murine brain is retained in the slices which, thus, represent a suitable three-dimensional matrix to study glioma cell invasion.

### Assessment of invasive capacity

**Fig. 3:**
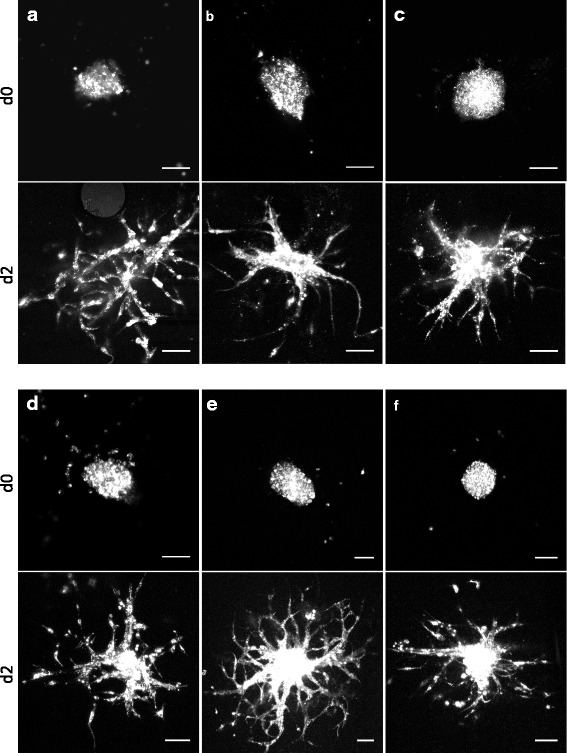
Improved ex vivo cell invasion assay. Representative confocal images of DiD labeled primary glioma cells (human (**a**), murine (**b**)) and established glioma cell lines (murine SMA560 (**c**) and human LN319 (**d**), U87MG (**e**) and U251MG (**f**)) implanted in adult brain slice cultures. Images were acquired at d0 (top) and d2 (bottom). Scale bars 100 μm, image quality was optimized by adjustment of brightness, contrast and gamma

To demonstrate that our ex vivo invasion assay protocol allows a high degree of glioma cell invasion, we manually implanted a panel of DiD labeled human and murine glioma spheroids into adult brain slices that had been adapted to in vitro conditions for 2 days. 48 h after implantation we fixed the slices and performed confocal imaging. As depicted in Fig. 3 all types of implanted glioma cells invaded strongly into the surrounding tissue. These observations suggest that the invasive capacity of different tumor cells can be reliably assessed and compared using this ex vivo invasion assay protocol.

### DiD labeling of tumor cells and optional tissue clearing enables high-contrast imaging

**Fig. 4:**
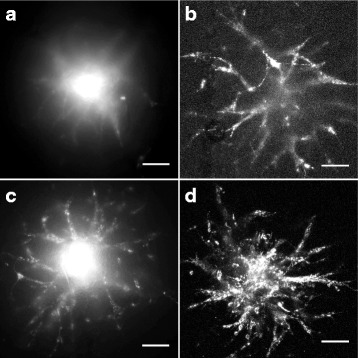
Improved image quality by DiD labeling and confocal imaging. Representative pictures of epi-fluorescence (**a**, **c**) and confocal microscopy (**b**, **d**). Usage of DiD (**c**, **d**) improves picture quality compared to DiI labeling of SMA560 cells (**a**, **b**). Scale bars 100 μm, image quality was optimized by adjustment of brightness, contrast and gamma

**Fig. 5:**
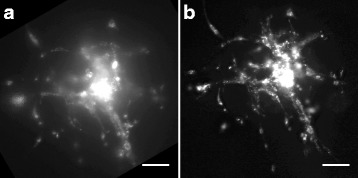
Improved epi-fluorescence microscopy after tissue clearing. DiD labeled LN319 cells imaged by epi-fluorescence microscope before (**a**) and after tissue clearing (**b**). Scale bars 100 μm, image quality was optimized by adjustment of brightness, contrast and gamma

Although previous studies have used ectopic GFP expression or the carbocyanine dye DiI for membrane labeling and tracing of cell invasion [13, 14, 16], we experienced high autofluorescence of the brain slice and a poor contrast between tissue and tumor cells when imaged with short excitation/emission wavelengths. To reduce this autofluorescent background we used the lipophilic carbocyanine dye DiD, an analog of DiI with markedly red-shifted fluorescence excitation and emission spectra. As autofluoresence decreases dramatically at longer wavelengths, DiD labeling resulted in strikingly sharper images compared to DiI (Fig. 4) and is moreover preferable for live cell imaging applications due to reduced photodamaging effects. To further improve epi-fluorescence imaging we cleared the brain tissue according to the SeeDB protocol [17]. As shown in Fig. 5 the quality of epifluorescence images of cleared brain slices was dramatically improved when compared to uncleared tissue. Taken together, our results show that tumor cell labeling with the carbocyanine dye DiD and brain slice clearing produce high-contrast epi-fluorescence images, representing a comparable alternative to confocal microscopy.

### Brain slices prepared from genetically engineered mice are suitable to study the stromal impact on tumor cell invasion

**Fig. 6:**
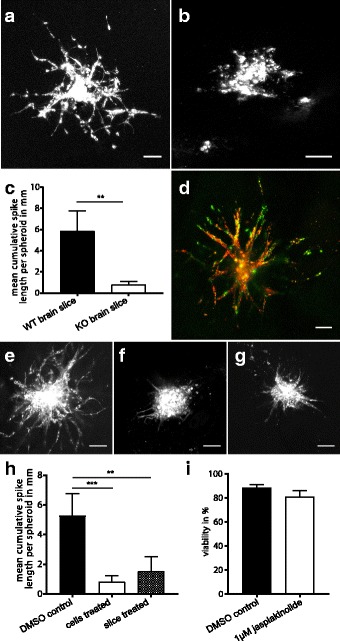
Multiple applications of the ex vivo invasion assay. Representative confocal images, scale bars 100 μm, image quality was optimized by adjustment of brightness, contrast and gamma. **a**-**c** Brain slices of genetically modified mice as a tool to modulate the microenvironment. Human glioma cell line LN319 implanted in **a** wild-type and **b** knockout brain slices. The deletion of a specific cell surface protein in the microenvironment significantly inhibits tumor cell invasion as quantified in (**c**). Error bars represent 95% confidence interval, (A) *n* = 7; (B) *n* = 5; **p < 0.001, Welch’s t-test. **d** Co-implantation of different cell types. Reactive astrocytes (pseudocolored in green, DiI labeled) and the murine glioma cell line SMA560 (red, DiD labeled) were co-cultured in a ratio of 3:2 as heterotypic multicellular spheroids and implanted in a wild-type brain slice. **e**-**i** The ex vivo invasion assay as a tool to identify invasion modulating compounds. 500 SMA560 glioblastoma cells were seeded per well to induce spheroid growth. 18 h prior to implantation, spheroids or brain slices were treated with 1 μM jasplakinolide. 24 h after implantation, brain slices were fixed and imaged by confocal microscopy. In contrast to **e** the highly invasive control-treated SMA560, **f** the treatment of the tumor cells or **g** brain slices with jasplakinolide significantly reduced their ability to invade without inducing cell death as examined by trypan blue staining (**i**). **h** Quantification of invasion. Error bars show 95% confidence interval, **e** *n* = 12; **f**, **g** n = 7; **i** *n* = 3; **p < 0.001; ***p ≤ 0.0001; Welch’s t-test, *p*-values Bonferroni corrected

The influence of the microenvironment on the invasive behavior of tumor cells has been increasingly recognized. To illustrate that the ex vivo invasion assay enables the study of tumor cell/microenvironment interactions, we used brain slices prepared from a genetically engineered mouse with a global loss of a cell surface protein. We observed a substantial reduction in tumor cell invasion upon spheroid implantation in knockout compared to control wild-type brain slices (Fig. 6a-c). Thus, our results demonstrate the suitability of this assay to study the impact of stromal components on tumor cell invasion by using brain slices from genetically engineered mice.

### Heterotypic spheroids allow direct comparison of different cell types and their mutual influence on invasion

To examine the mutual influence of different cell types on their invasive properties, we performed implantations of heterotypic spheroids composed of tumor cells and other cell types differentially labeled with fluorescent dyes. In heterotypic spheroids generated from tumor cells and reactive astrocytes, we observed migration of both cell types (Fig. 6d) without identifying one cell type as the leading cell. Similarly, this method allows for the co-cultivation of control and genetically modified tumor cells, i.e. harboring gain or loss of specific proteins, and direct comparison of their invasive behavior.

### The ex vivo invasion assay as a tool to identify small molecules affecting invasion

Finally, we evaluated whether the system is suitable for small molecule treatment for the testing of cell invasion modulating compounds. In our experimental setting the drug can be applied either directly to the tumor cells or to the environment. As a proof of principle, we treated tumor spheroids before implantation with jasplakinolide, a known inhibitor of migration [25, 26]. Previously, an inhibitory effect of 1 μM jasplakinolide on actin depolymerization and thus cell migration had been described [26, 27]. Consistent with this, we observed significantly less tumor cell invasion upon tumor cell treatment with 1 μM jasplakinolide for 18 h (Fig. 6e, f, h). Importantly, cell death was not induced under these conditions (Fig. 6i). We obtained similar results when treating the brain slice with 1 μM jasplakinolide 18 h prior to implantation (Fig. 6g). This result highlights the application of our method to drug discovery and preclinical evaluation, by permitting the selection of compounds affecting tumor cell invasion prior to in vivo testing.

## Discussion

In the past decades tumor cell invasion has primarily been assessed by inexpensive and rapid two-dimensional assays. However, these cell culture models are very limited in their power to accurately predict the effect of proteins or small molecules on cell invasion in vivo, probably due to functional differences of proteins between two- and three-dimensional migration and the absence of environmental influences [28]. Although animal models are thought to represent the most reliable method of investigating cell invasion, they involve not only high cost but also ethical and technical concerns. The laborious and time inefficient application of mouse models is especially disadvantageous for co-clinical and personalized treatment studies that rely on a rapid read out and the exclusion of false-positive candidate compounds identified by conventional two-dimensional assays [29]. This has resulted in attempts to bridge the gap between over-simplified cell culture approaches and the more meaningful, but inefficient, in vivo models with reproducible ex vivo techniques. The current state of the art to mimic the natural environment of glioma cells are organotypic brain slice cultures that can be cultivated ex vivo for several days to weeks without considerable loss of their cytoarchitecture. Retaining their physiological structure, brain slices provide an optimal three-dimensional matrix for ex vivo invasion assays. To our knowledge, mostly perinatal donors have been used for the preparation of organotypic brain slices due to their high mechanical and ischemic resistance. However, in rodents the ECM is substantially remodeled starting from 2 weeks after birth. This remodeled and thus significantly firmer ECM is subsequently maintained throughout adulthood [30]. Similarly, myelination of nerve fibers occurs predominantly postnatally and can be extended to adulthood [31]. Hence, absent or incomplete myelination and the immature and loose extracellular matrix are profound differences between neonatal and mature adult brain tissue. In order to reflect the age related disease of adult glioma, we decided to use adult brain slices that exhibit a mature myelination pattern and ECM composition.

While there are previous reports that use organotypic brain slices for tumor cell invasion assessment, we still lack a simple, standardized, and reproducible protocol that allows its application in basic and preclinical research. Various approaches for the co-cultivation of tumor cells and slices and the measurement of cell invasion have been published. For brain metastasis research, tumor cells have been seeded in a matrigel plug adjacent to the brain slice in order to investigate interactions between cancer and glial cells by fluorescence microscopy [32]. However, this model is unsatisfactory when used to examine the invasion of primary brain tumor cells, as they arise and migrate within the brain tissue and moreover do not encounter an environment comparable to matrigel. Other publications have developed this approach by seeding single cells on top of the slices [33]. However, we see disadvantages in this technique, as it is disturbed by the diffusion of single cells after seeding and requires life cell imaging, preferably by an upright confocal microscope, which is not universally available. Moreover, we have observed reduced invasion when the tumor cells were seeded on top instead of within the tissue, as the cells migrate on the slice surface instead of efficiently penetrating the tissue. In contrast, we implant tumor cells as spheroids within the tissue in order to position them at a specific location of the brain slice and to provide a comparable starting point for the assay. Indeed, other publications have reported this approach; however, they show a low grade of invasion that does not reflect the aggressive infiltration observed in patients [13, 15, 16]. Following our protocol, we could observe strong invasion in a panel of human and murine glioma cell lines as well as primary cells. Moreover, we could improve the imaging quality of implanted tumor cells using DiD, which drastically reduces autofluorescence compared to the commonly applied DiI. Epi-fluorescence microscopy was further optimized by tissue clearing according to the SeeDB protocol [17]. Thus, we have generated an optimized and standardized protocol that acts as the basis of a set of functional applications, some of which we describe here. The possibility of specifically manipulating either one or both compartments involved in tumor cell migration, the microenvironment and the tumor cells themselves, and to monitor the consequences of the manipulation on tumor cell invasion is a major advantage of this improved protocol. Here, we demonstrate the flexibility of the protocol by manipulating the microenvironment with exogenous small molecule treatment or using genetically modified mice as donors for the organotypic brain slice cultures. This allows the investigation of the effects of global inactivation of a gene or gene product of interest, as well as cell type-specific deletion or overexpression, depending on the mouse model used. In addition, this assay is suitable for direct manipulation of the tumor cells themselves by gain- or loss-of-function approaches. Consequently, the combination of both strategies is a powerful tool i) to identify critical factors in tumor cells and their putative interaction partners in the tumor microenvironment and ii) to dissect their mode of action in tumor cell invasion, ultimately yielding potential novel targets for brain tumor therapy.

## Conclusion

As growing evidence suggests the tumor microenvironment as a key factor in tumor cell invasion, we optimized an ex vivo invasion assay that can reliably and quantitatively measure glioma cell movement in an environment that more accurately recapitulates the physiological state in vivo. The functional applications described demonstrate the power and versatility of this method as an advance over previous work, and will encourage the use of more relevant models than traditional two-dimensional migration assays.

## Abbreviations

DiD: 1,1′-dioctadecyl-3,3,3′,3′- tetramethylindodicarbocyanine, 4-chlorobenzenesulfonate salt
DiI: 1,1′-dioctadecyl-3,3,3′,3′-tetramethylindocarbocyanine
DMEM: Dulbecco’s Modified Eagle Medium
DMSO: Dimethyl sulfoxide
ECM: extracellular matrix
EGF: Epidermal growth factor
FGFb: Basic fibroblast growth factor
GFAP: glial fibrillary acidic protein
GFP: Green fluorescent protein
HEPES: 4-(2-hydroxyethyl)-1-piperazineethanesulfonic acid buffer
Iba1: ionized calcium-binding adapter molecule 1
MBP: myelin basic protein
MEM: Minimum Essential Medium
PBS: Phosphate Buffered Saline
